# Once‐weekly glucagon‐like peptide receptor agonist polyethylene glycol loxenatide protects against major adverse cardiovascular events in patients with type 2 diabetes: a multicenter ambispective cohort study (FLYING trial)

**DOI:** 10.1002/mco2.70094

**Published:** 2025-02-13

**Authors:** Jilin Li, Yu Tian, Liping Li, Yanyan Zhao, Shuhui Yang, Wencan Xu, Dan Zhu, Junjun Ye, Jingxian Chen, Weiting Liu, Haibo Xue, Wei Wu, Feiying Deng, Yale Duan, Zhizhen Hu, Bin Xie, Zhe‐Sheng Chen, Kaijian Hou

**Affiliations:** ^1^ School of Public Health Shantou University Shantou China; ^2^ Department of Cardiovascular The Second Affiliated Hospital of Medical College of Shantou University Shantou China; ^3^ Research Center Huizhou Central People's Hospital Guangdong Medical University Huizhou China; ^4^ School of Public Health Benedictine University Lisle Illinois USA; ^5^ Department of Endocrinology and Metabolism The First Affiliated Hospital of Zhengzhou University Zhengzhou China; ^6^ Department of Endocrine and Metabolic Diseases Shantou Central Hospital Shantou China; ^7^ Department of Endocrinology The First Affiliated Hospital of Shantou University Medical College Shantou China; ^8^ Graduate school Shantou University Medical College Shantou China; ^9^ School of Nursing Anhui University of Chinese Medicine Anhui China; ^10^ Department of Endocrinology and Metabolism Binzhou Medical University Hospital Binzhou China; ^11^ Guangdong Provincial Institute of Public Health Guangdong Provincial Center for Disease Control and Prevention Guangzhou China; ^12^ Department of Medical Affairs Jiangsu Hansoh Pharmaceutical Group Co., Ltd. Shanghai China; ^13^ Department of Pharmaceutical Sciences Institute for Biotechnology College of Pharmacy and Health Sciences St. John's University Queens New York USA

**Keywords:** ambispective cohort study, cardiovascular, glucagon‐like peptide‐1 receptor agonist, myocardial infarction, polyethylene glycol loxenatide, type 2 diabetes

## Abstract

This study aimed to determine the effects of polyethylene glycol loxenatide (PEG‐Loxe), a glucagon‐like peptide‐1 receptor agonist, on a three‐point major adverse cardiovascular event (3P‐MACE) in patients with type 2 diabetes mellitus (T2DM). The study was conducted in six tertiary hospitals in three cities in China. Large language models were used to retrospectively screen and include 12,341 patients with T2DM who had either cardiovascular disease or cardiovascular risk factors. The patients were divided into the PEG‐Loxe cohort (treated with PEG‐Loxe, *n* = 1282) and the control cohort (treated with incretin glucose‐lowering agents, *n* = 11,059). After a median follow‐up of 4.0 years, 3P‐MACE occurred in 51 (4.0%) and 1263 (11.4%) patients in PEG‐Loxe and control cohorts, respectively (hazard ratio [HR] 0.68, 95% confidence interval [CI] 0.49–0.94; *p *= 0.019). In the PEG‐Loxe versus control cohorts, 21 (1.6%) versus 476 (4.3%) patients experienced nonfatal stroke (HR 0.63; *p *= 0.041), whereas 22 (1.7%) versus 545 (4.9%) experienced nonfatal myocardial infarction (HR 0.66; *p *= 0.058), and the incidence of cardiovascular death was 8 (0.6%) versus 240 (2.2%), respectively (HR 0.56; *p *= 0.118). We found a significantly lower incidence of first nonfatal myocardial infarction, nonfatal stroke, or cardiovascular deaths in the PEG‐Loxe cohort than the control cohort.

## Graphical abstract

Large language models were used to retrospectively screen and include 12,341 patients with T2DM who had either cardiovascular disease or cardiovascular risk factors. We found a significantly lower incidence of first nonfatal myocardial infarction, nonfatal stroke, or cardiovascular deaths in the PEG‐Loxe cohort than the control cohort.

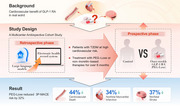

## 1 INTRODUCTION

Diabetes mellitus (DM) is a chronic metabolic disease characterized by persistent chronic hyperglycemia, with a global prevalence of 10.5% and projections suggesting that this number will rise to 783 million by 2045.^1^ According to the International Diabetes Federation, approximately 537 million adults worldwide had diabetes in 2021, with 6.7 million deaths related to diabetes or its complications. Prolonged hyperglycemia can damage major and micro blood vessels, endangering the heart, brain, kidneys, and other organs.^2^ Particularly, cardiovascular disease (CVD) accounts for over half of diabetes‐related deaths.^3^

Managing cardiovascular complications has been a significant challenge in diabetes treatment. The advent of novel glucagon‐like peptide‐1 receptor agonists (GLP‐1RA) and sodium‐glucose cotransporter‐2 (SGLT2) inhibitors has shown promise in improving the clinical outcomes of patients with diabetes.^4^ GLP‐1RA has partial or complete amino acid sequence homology with glucagon‐like peptide‐1 (GLP‐1), which is released by L cells of intestine.^5^ GLP‐1RA mimics the action of the natural GLP‐1 and plays a role in diabetes treatment by acting on the GLP‐1 receptor. However, unlike the natural GLP‐1, GLP‐1RA is not easily degraded by enzymes; therefore, its effect lasts longer and can better meet the needs of clinical treatment.^6^ GLP‐1RAs exert glucose‐lowering effects through multiple pathways, including increasing insulin secretion, inhibition of glucose secretion in a glucose‐dependent manner, appetite suppression, slowing of gastric emptying, promotion of satiety, improvement of insulin sensitivity, and inhibiting hepatic glucose output.^7^ Importantly, several large studies on cardiovascular outcomes have demonstrated that some GLP‐1RAs (dulaglutide, liraglutide, semaglutide, albiglutide, and efpeglenatide) reduce the risk of major adverse cardiovascular events (MACE) in patients with type 2 diabetes mellitus (T2DM) and improve their clinical outcomes.^8^, ^9^, ^10^, ^11^, ^12^ In addition, double or triple‐target drugs based on polypharmacology are being developed to improve the efficacy of GLP‐1 agonists, such as tirzepatide, whose effects on cardiovascular risk are also being studied.^13^, ^14^ However, in clinical practice, the effect of GLP‐1RA on cardiovascular outcomes in patients with T2DM is unclear.

Polyethylene glycol loxenatide (PEG‐Loxe) is a once‐weekly GLP‐1RA formulation approved by China in May 2019. The structure of PEG‐Loxe differs from the human GLP‐1‐derived dulaglutide, liraglutide, and semaglutide. PEG‐Loxe is derived from exendin‐4 by substituting four amino acids and linking them to a PEG molecule.^15^ The half‐life of PEG‐Loxe is 131.8–139.8 h.^16^ The efficacy and safety of PEG‐Loxe have been verified in previous studies,^17^, ^18^ and it has been found to significantly reduce blood glucose and body weight in patients with T2DM and improve blood lipids and blood pressure compared with the control group.^19^, ^20^ A recent study showed that PEG‐Loxe reduced endothelial progenitor cell apoptosis.^21^ Another study showed that PEG‐Loxe could improve endothelial cell function in middle‐aged and elderly patients with T2DM.^22^ In addition, PEG‐Loxe can improve the urinary protein levels in patients with mild to moderate diabetic kidney disease.^20^ However, the role of PEG‐Loxe on cardiovascular complications in patients with T2DM remains unclear, and relevant studies are lacking.

Several guidelines recommend the use of GLP‐1RA in patients with T2DM who have cardiovascular risk factors due to proven cardiovascular benefits, but the use of PEG‐Loxe has been limited.^23^, ^24^ To address this gap, this study aimed to determine the effects of PEG‐Loxe on a three‐point major adverse cardiovascular event (3P‐MACE) in patients with T2DM high cardiovascular risk in a real‐world setting. This research seeks to provide valuable insights into the cardiovascular effects of PEG‐Loxe and its role in managing cardiovascular risks in patients with T2DM.

## 2 RESULTS

### 2.1 Patients’ baseline characteristics

**FIGURE 1:**
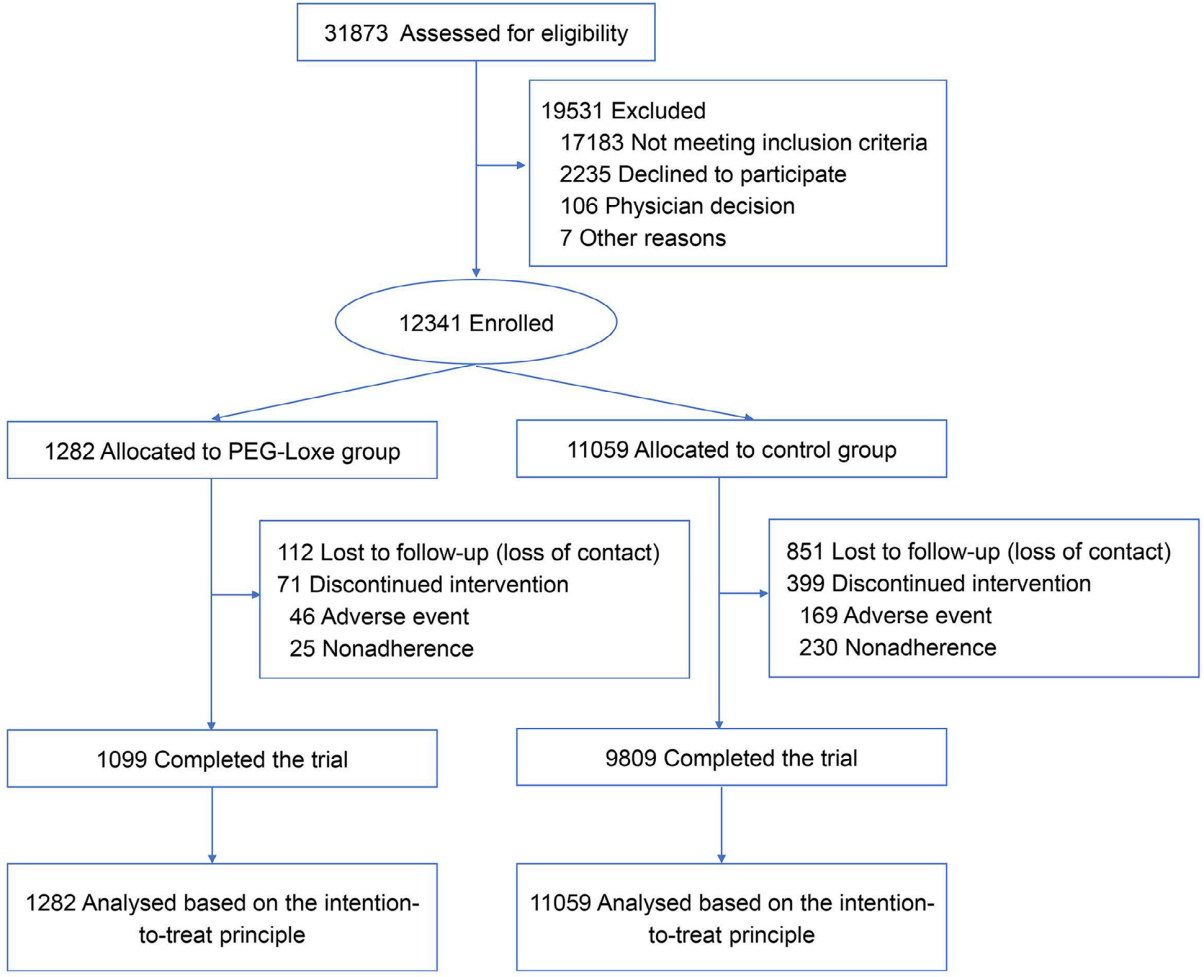
Flow diagram of the study. PEG‐Loxe, polyethylene glycol loxenatide.

Among the 31,873 patients with T2DM who had at least one CVD or one cardiovascular risk factor, 12,341 met the inclusion criteria and were enrolled in this study (Figure 1). Among the 12,341 patients, 1282 were treated with weekly subcutaneous injections of 0.2 mg PEG‐Loxe (the PEG‐Loxe cohort). The remaining 11,059 patients (control cohort) were treated with non‐incretin‐based therapies (besides GLP‐1RA and dipeptidyl peptidase‐4 inhibitors). As of December 31, 2023, the participants were followed up for a median period of 4.0 years, with a total follow‐up of 42,523.8 person‐years.

The baseline characteristics of the patients are shown in Table 1. Patients in the PEG‐Loxe and control cohorts had a mean age of 63.3 and 65.6 years, a mean body mass index (BMI) of 26.9 and 24.8 kg/m^2^, and 42.4% and 47.1% were females, respectively. Approximately half the patient population in the PEG‐Loxe and control groups had a history of CVD (47.5% and 48.8%, respectively) and were treated with SGLT2 inhibitors (8.3% and 20.1%, respectively).

**TABLE 1:** Baseline characteristics.

Characteristic	PEG‐Loxe (*n* = 1282)	Control (*n* = 11,059)
Age, years	63.3 (15.0)	65.6 (10.6)
Female, *N* (%)	543 (42.4)	5212 (47.1)
HbA1c, %	8.54 (1.25)	8.48 (3.15)
BMI, kg/m^2^	26.9 (1.6)	24.8 (3.7)
Duration of T2DM, years	9.7 (5.7)	10.5 (6.1)
CVD history, *N* (%)	609 (47.5)	5394 (48.8)
SBP, mmHg	138.1 (16.2)	138.8 (16.5)
DBP, mmHg	80.0 (11.7)	80.7 (11.8)
TC, mmol/L	4.94 (1.04)	4.89 (1.67)
TG, mmol/L	2.12 (1.82)	2. 07 (2.51)
HDL‐C, mmol/L	1.21 (0.32)	1.11 (0.36)
LDL‐C, mmol/L	2.91 (0.80)	3.11 (1.16)
Smoke, *N* (%)	137 (10.7)	1108 (10.0)
CKD, *N* (%)	470 (36.6)	3846 (34.8)
Diabetic retinopathy, *N* (%)	96 (7.5)	755 (6.8)
History of hypertension, *N* (%)	1062 (82.8)	9249 (83.6)
Metformin, *N* (%)	590 (46.0)	4574 (41.4)
SU/GLN, *N* (%)	66 (5.1)	1311 (11.9)
AGI, *N* (%)	444 (34.6)	3716 (30.8)
TZD, *N* (%)	91 (7.1)	738 (6.7)
SGLT2i, *N* (%)	107 (8.3)	2220 (20.1)
Insulin, *N* (%)	417 (32.5)	5970 (54.0)
ACEI/ARB, *N* (%)	821 (64.0)	6781 (61.3)
Beta‐blocker, *N* (%)	435 (33.9%)	4302 (38.9)
Statin, *N* (%)	803 (62.6)	6799 (61.2)
Fibrate, *N* (%)	79 (6.2)	782 (7.1)
Antiplatelet, *N* (%)	619 (48.3)	5559 (50.3)

Abbreviations: AGI, alpha‐glucosidase inhibitors; ACEI/ARB, angiotensin‐converting enzyme inhibitors/angiotensin II receptor blockers; BMI, body mass index; CVD, cardiovascular disease; DBP, diastolic blood pressure; GLN, glinides; HDL‐C, high‐density lipoprotein cholesterol; HbA1c, glycated hemoglobin; LDL‐C, low‐density lipoprotein cholesterol; PEG‐Loxe, polyethylene glycol loxenatide; SU, sulfonyl ureas; SBP, systolic blood pressure; SGLT2i, sodium–glucose cotransporter‐2 inhibitors; TC, total cholesterol; TG, triglyceride; TZD, thiazolidinediones.

**FIGURE 2:**
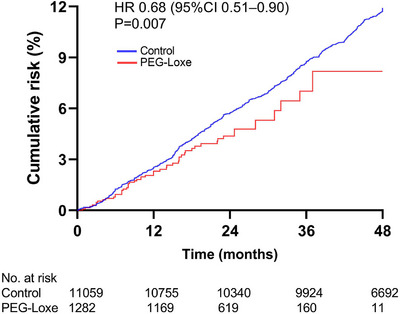
Cumulative incidence of three‐point major adverse cardiovascular event (3P‐MACE). HR, hazard ratio; PEG‐Loxe, polyethylene glycol loxenatide.

The primary outcome (3P‐MACE) was observed in 51 (4.0%) patients in the PEG‐Loxe cohort and 1263 (11.4%) patients in the control cohort, corresponding to a rate of 2.32 per 100 person‐years for the PEG‐Loxe group and 3.13 per 100 person‐years for the control group (hazard ratio [HR] 0.68, 95% confidence interval [CI] 0.49–0.94, *p*‐value = 0.019) (Figure 2 and Table 2). HRs were adjusted for potential confounding factors, including baseline demographics, comorbidities, laboratory results, co‐medication, and the centers where patients were treated. The multivariate Cox regression model is shown in Table S1.

**TABLE 2:** Primary and secondary outcomes.

	PEG‐Loxe (*n* = 1282)		Control (*n* = 11,059)			
Event	No. of participants (%)	No. of events/100 person‐years	No. of participants (%)	No. of events/100 person‐years	Hazard ratio (95% CI)	*p* value
3P‐MACE	51 (4.0)	2.32	1263 (11.4)	3.13	0.68 (0.49–0.94)	0.019
Expanded MACE	66 (5.1)	3.01	1660 (15.0)	4.12	0.72 (0.56–0.93)	0.012
Myocardial infarction	25 (2.0)	1.14	584 (5.3)	1.45	0.69 (0.46–1.04)	0.078
Nonfatal myocardial infarction	22 (1.7)	1.00	545 (4.9)	1.35	0.66 (0.43–1.01)	0.058
Fatal myocardial infarction	3 (0.2)	0.14	39 (0.4)	0.10	0.94 (0.27–3.29)	0.922
Stroke	23 (1.8)	1.05	533 (4.8)	1.32	0.65 (0.43–1.00)	0.051
Nonfatal stroke	21 (1.6)	0.96	476 (4.3)	1.18	0.63 (0.40–0.98)	0.041
Fatal stroke	2 (0.2)	0.09	57 (0.5)	0.14	0.53 (0.12–2.24)	0.384
CV death	8 (0.6)	0.36	240 (2.2)	0.60	0.56 (0.27–1.16)	0.118
Coronary revascularization	36 (2.8)	1.64	878 (7.9)	2.18	0.80 (0.57–1.13)	0.202
Hospitalization for unstable angina	7 (0.5)	0.32	143 (1.3)	0.35	0.81 (0.37–1.76)	0.590
Hospitalization for heart failure	8 (0.6)	0.36	243 (2.2)	0.60	0.79 (0.39–1.63)	0.529
All‐cause death	18 (1.4)	0.82	538 (4.9)	1.33	0.66 (0.41–1.08)	0.097

Abbreviations: CI, confidence interval; 3P‐MACE, three‐point major adverse cardiovascular event; PEG‐Loxe, polyethylene glycol loxenatide, CV, cardiovascular.

The incidence of primary and secondary outcomes in the PEG‐Loxe and control cohorts are shown in Table 2.

The incidences of coronary revascularization, hospitalization for unstable angina, hospitalization for heart failure (HF), and all‐cause mortality were slightly lower in the PEG‐Loxe cohort, but the differences were not statistically significant (Table 2).

**FIGURE 3:**
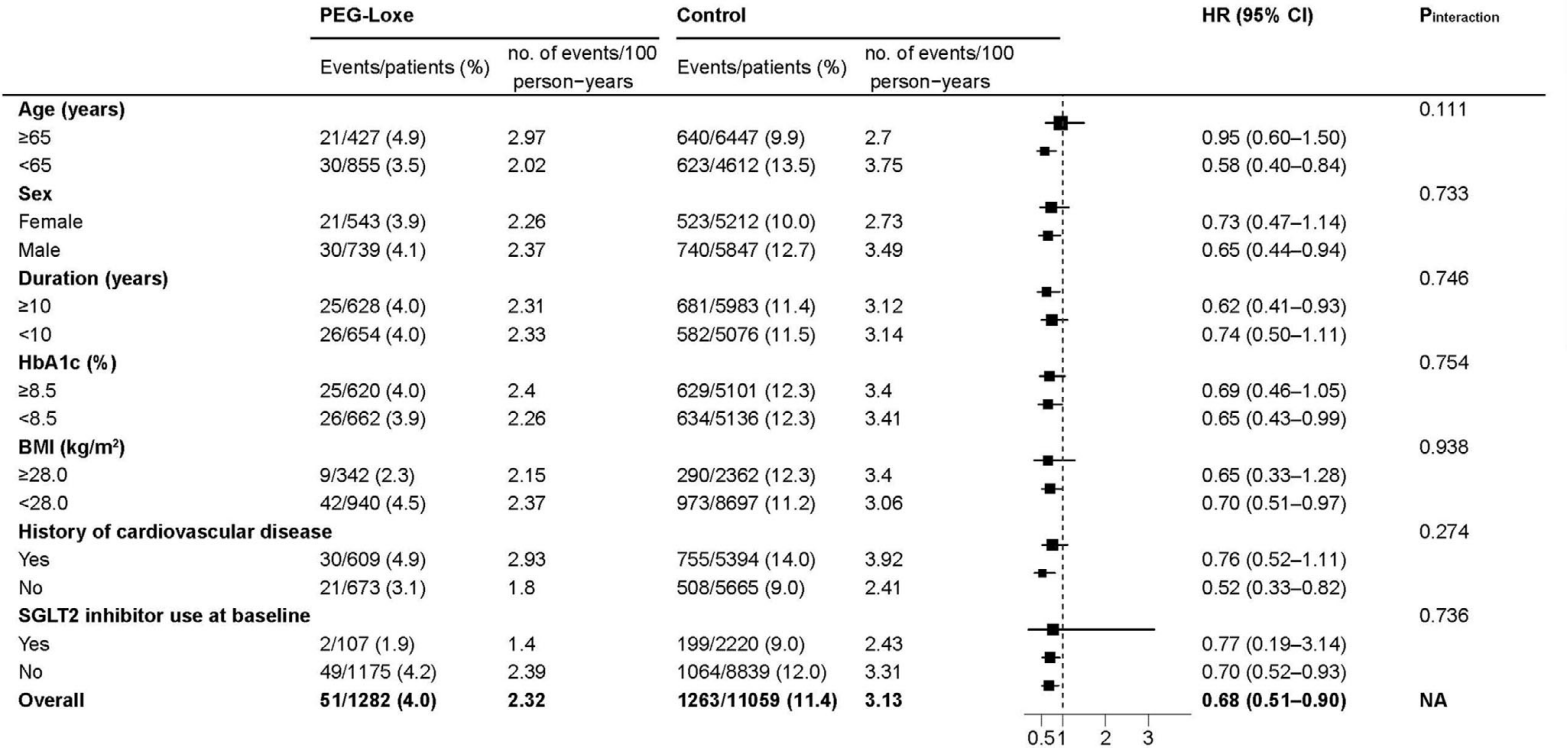
Subgroup analyses for three‐point major adverse cardiovascular event (3P‐MACE). BMI, body mass index; HR, hazard ratio; NA, not applicable; PEG‐Loxe, polyethylene glycol loxenatide; SGLT2, sodium‐glucose cotransporter‐2.

Subgroup analysis of the primary endpoints revealed no significant interactions among age, sex, duration of T2DM, glycated hemoglobin (HbA1c), BMI, history of CVD, or use of SGLT2 inhibitors (Figure 3). The mean change in HbA1c was −1.05% (95% CI: −1.16 to −0.93) in the PEG‐Loxe cohort and −0.87% (95% CI: −0.91 to −0.83) in the control cohort (*p *= 0.004). The mean change in body weight was −2.16 kg (95% CI: −2.51 to −1.82) in the PEG‐Loxe cohort and 1.01 kg (95% CI: 0.89 to 1.13) in the control cohort (*p *< 0.001).

The percentage of patients who reported severe gastrointestinal events was higher in the PEG‐Loxe cohort than in the control cohort (*p *= 0.016). Other prespecified safety outcomes were similar between the two cohorts (Table 3).

**TABLE 3:** Adverse events of special interest.

Adverse events	PEG‐Loxe No. of participants (%)	Control No. of participants (%)	*p* value
Severe gastrointestinal event	22 (1.7)	109 (1.0)	0.021
Diabetes‐related blindness	3 (0.2)	27 (0.2)	0.620
Acute pancreatitis	17 (1.3)	129 (1.2)	0.682
Any cancer	40 (3.1)	387 (3.5)	0.519
Thyroid c‐cell neoplasm	0 (0)	0 (0)	1.000
Acute kidney failure	18 (1.4)	175 (1.6)	0.640
Severe hypoglycemia	4 (0.3)	47 (0.4)	0.654

Abbreviations: PEG‐Loxe, polyethylene glycol loxenatide.

Sensitivity analysis showed the robustness of the primary outcome results. We used the stepwise model to select covariates with *p* < 0.05. Various propensity score matching (PSM) approaches were tested, including nearest matching with 1:1 and 1:2 ratios, calipers set at 0.1 and 0.2, and optimal matching. Among these methods, the caliper of 0.2 demonstrated superior performance, as it effectively balanced covariates with a standardized mean difference close to 0 while retaining the largest number of patients. The characteristics of the cohort matched with a caliper of 0.2 are detailed in Table S2. Welch's two‐sample *t*‐test revealed a statistically significant difference in the mean 3P‐MACE between the two groups (*p* < 0.001). In the adjusted multivariate Cox model applied to the propensity score‐matched data, the HR for 3P‐MACE was 0.63 (95% CI: 0.43–0.91; *p* = 0.015) (Table S3). The results were similar to the primary analysis. The results support the conclusion that there is a significant difference in the occurrence of 3P‐MACE between the two treatment groups, with the control group experiencing a higher mean number of events compared to the PEG‐Loxe group.

## 3 DISCUSSION

In this multicenter ambispective cohort study, it was found that PEG‐Loxe was associated with a decreased risk of MACE by 32% compared with the use of non‐incretin anti‐diabetic drugs in patients with T2DM who had a history of CVD or a cardiovascular risk factor. Thus, an estimated 14 patients would need to be treated with PEG‐Loxe for 4.0 years to prevent one MACE.^25^ This cardiovascular benefit manifested primarily as a significant reduction in the risk of nonfatal stroke (37%). Most study participants were treated with anti‐hypertensive, lipid‐lowering, and antiplatelet agents at baseline.

In the adjusted multivariate Cox model of PSM data, the HR of 3P‐MACE was 0.63, which is close to the HR of 0.68 in the primary analysis, suggesting a potentially more pronounced effect in the matched analysis. The reasons for the discrepancy include (1) PSM balances treatment groups with respect to observed covariates, reducing bias and providing a more accurate estimate of the treatment effect; (2) the smaller matched dataset (2228 patients) may yield different estimates compared to the larger original dataset, which may dilute the effect or introduce variability; (3) differences in covariate adjustments between models can impact HR estimates; and (4) the primary analysis may not fully account for all confounding factors addressed by the matched analysis. The difference in HR indicates that the PSM analysis provides a better perspective on the treatment effect, suggesting that the primary analysis may have been influenced by covariate imbalances or confounding factors (Table S6).

GLP‐1RA is derived by modifying GLP‐1 or exendin‐4. Is the cardiovascular benefit of GLP‐1RA a drug‐class effect? Liraglutide, semaglutide, and dulaglutide are derived from GLP‐1 with cardiovascular benefits.^8^, ^9^, ^10^ In contrast, lixisenatide and exenatide are derived from exendin‐4, and they have been shown to have a safe cardiovascular profile.^11^, ^26^, ^27^ A clinical investigation report on patients with T2DM with CVD and/or a cardiovascular risk factor found that efpeglenatide (derived from exendin‐4) treatment for 1.8 years reduced the probability of 3P‐MACE by 27%.^11^ PEG‐Loxe is also derived from exendin‐4.^16^ The present study enrolled patients with high cardiovascular risk and reported that treatment regimens including PEG‐Loxe significantly reduced the risk of 3P‐MACE. Our study findings support the hypothesis that the cardiovascular benefits of GLP‐1RA are a drug‐class effect.

Pan et al. suggested that the cardiovascular benefits of incretin‐based therapy may occur at higher doses of GLP‐1RAs and that cardiovascular benefits not observed in some GLP‐1RAs may be related to inadequate drug exposure.^28^ With a *C*_max_ of 38.5 pmol/L for lixisenatide^29^ and 71.4 pmol/L for exenatide,^30^ no cardiovascular benefit was observed.^26^, ^27^ Liraglutide, semaglutide, and dulaglutide had *C*_max_ of 22,000,^31^ 30,000,^32^ and 1,810 pmol/L,^33^ respectively, and cardiovascular benefits were observed.^8^, ^9^, ^10^ Similarly, loxenatide has a *C*_max_ of 20,161 pmol/L.^16^ The cardiovascular benefit of loxenatide observed in this study may be related to its higher drug exposure.

Our study design differed from those of previous GLP‐1RA trials on cardiovascular outcomes because all the other previous studies used a randomized controlled trial (RCT) study design. Although the RCT design in trials on cardiovascular outcomes can minimize possible bias, balance confounding factors, and improve statistical test efficacy,^34^ the potential ethical problems with control group interventions, as well as the time and economic costs, limit the implementation of RCTs. An ambispective cohort study is a combination of a prospective cohort study and a retrospective cohort study, that is, a retrospective cohort study followed by a prospective cohort study for a period of time. Compared to RCTs, ambispective cohort studies can leverage existing medical records and databases to collect large amounts of data faster and at a lower cost. This can simplify the research process, shorten the research period, and make the implementation of such research simpler and easier.

Real‐world data cover a wide range of populations, making the results of research and analysis based on these data more general and representative. This helps to reveal complex relationships and phenomena that are difficult to observe in idealized experimental settings. However, the quality of real‐world data can be affected by various factors, such as the accuracy, completeness, and consistency of data records. Large language models (LLMs), a cutting‐edge technology of artificial intelligence, are neural network models composed of billions of parameters. LLMs are trained using textual big data and are capable of compiling and extracting medical information from electronic health record systems.^35^ We used LLM to iterate learning and validation to screen predefined keywords and identify target patients from massive amounts of unstructured data. The use of this technology enabled the rapid screening of patients with T2DM who were eligible for inclusion in the study. This increases efficiency and reduces human error, thereby providing an effective tool for this type of clinical study.

The study had several limitations. First, missing data from retrospective studies is very common, which can introduce bias to the study results. Second, MACE is a medical emergency, and patients with MACE are usually sent to the nearest medical facility. Therefore, we acknowledge the incomplete collection of MACE data. Third, since there was no control for other covariates in this real‐world study, the effects of the combination of multiple medications on the observed benefits of PEG‐Loxe on cardiovascular risk factors cannot be ruled out. Therefore, the results must be interpreted with caution.

## 4 CONCLUSIONS

The incidence of the first occurrence of nonfatal myocardial infarction, nonfatal stroke, or cardiovascular death was significantly lower in patients with T2DM at high cardiovascular risk treated with PEG‐Loxe than those treated with non‐incretin‐based therapies.

## 5 MATERIALS AND METHODS

### 5.1 Design overview

This multicenter ambispective cohort study was conducted both retrospectively and prospectively. During the retrospective phase, patients with T2DM were identified based on the existing diagnosis in the electronic medical record system, and those meeting the inclusion criteria were screened between July 1, 2020, and June 30, 2023. Self‐reported T2DM was not included in the study. The baseline characteristics and data on cardiovascular events were collected. In the prospective phase, these patients were followed up through December 31, 2023, and data on cardiovascular events that occurred during the follow‐up period were collected. Patients were divided into a PEG‐Loxe (treated with PEG‐Loxe) and a control cohort (treated with non‐incretin‐based therapies) (Figure S1). The Institutional Review Board of the Second Affiliated Hospital of Shantou University Medical College approved the study protocol (approval number: 2023–39). The study was conducted in accordance with the principles of the Declaration of Helsinki and has been registered in the China Clinical Trial Registry (ChiCTR2300073001). The Institutional Review Board of the Second Affiliated Hospital of Shantou University Medical College waived the requirement to document informed consent as the study posed minimal risk to the participants, and the actions or procedures conducted would not require written informed consent if taken out of the context of this study.

### 5.2 Settings and participants

This study was conducted in six tertiary hospitals in three cities: The First Affiliated Hospital of Shantou University Medical College; The Second Affiliated Hospital of Medical College of Shantou University; Shantou Central Hospital; First Affiliated Hospital of Zhengzhou University; Binzhou Medical University Hospital; and Longhu People's Hospital.

We used LLMs to process unstructured clinical data in the electronic health record system.^35^ Briefly, unstructured data were collected and pooled from various centers. LLMs screened for predefined keywords through repeated learning and validation. This was followed by an initial screening of the patients who met the inclusion criteria in the system. Then, the unstructured data were transformed into structured data, followed by a manual review to avoid data errors. The index date was the date of the first prescription for PEG‐Loxe or non‐incretin‐based therapies. Drug exposure was calculated using the time between the first and last prescriptions.^36^

The inclusion criteria were as follows: (1) patients with T2DM; (2) treatment with PEG‐Loxe or non‐incretin‐based therapies for over 6 months; and (3) at least one CVD or one cardiovascular risk factor (Table S5).^37^ The coronavirus disease 2019 (COVID‐19) epidemic occurred within the study period.^38^ As COVID‐19 infection increases the risk of CVD,^39^ patients with T2DM hospitalized for COVID‐19 during the study period were excluded.

For patients who met the inclusion criteria, a 6‐month follow‐up was planned, with one visit every 3 months, making a total of two visits. Laboratory indicators, cardiovascular events, and adverse events in the electronic medical records system and during follow‐up visits were collected.

### 5.3 Study outcomes

The primary outcome was the first occurrence of 3P‐MACE (including nonfatal myocardial infarction, nonfatal stroke, or cardiovascular death). The secondary outcomes included an expanded MACE (including nonfatal myocardial infarction, nonfatal stroke, cardiovascular death, coronary revascularization, or hospitalization for unstable angina) and its components, hospitalization for HF, and all‐cause death. These outcomes were coded according to the International Classification of Diseases‐10 codes (Table S6). Coronary revascularization was defined as coronary artery bypass grafting and percutaneous coronary intervention.

All glucose‐lowering drugs used in this study have been approved and are available in the market. Regarding the safety endpoints, several adverse events of special interest were observed, including serious gastrointestinal events, diabetes‐related blindness, acute pancreatitis, any cancers, medullary thyroid carcinoma, acute kidney failure, and severe hypoglycemia. The safety endpoints are defined in Table S6.

### 5.4 Statistical analysis

Although this was an observational study, primary and secondary endpoints were analyzed based on the intention‐to‐treat principle. Continuous variables are expressed as mean (standard deviation) or median (interquartile range) depending on whether they are normally distributed. Categorical variables are expressed as counts and percentages. Incidence rates were summarized as the number of events per 100 person‐years. For each event analysis, censored data were defined as cases where the event of interest had not occurred (e.g., death and loss to follow‐up) by December 31, 2023, which marked the end of the observation period. Kaplan−Meier curves were used to visualize the cumulative risk. Cox proportional risk models were then applied to estimate HRs and 95% CIs for primary and secondary endpoints. The model was adjusted for potential confounders, including baseline demographics, comorbidities, laboratory results, co‐medication, and the centers where patients were treated. Schoenfeld residuals test was employed to assess the proportional hazards assumption of the Cox models.

PSM was used for sensitivity analysis. PSM analysis was conducted to achieve balance across significant covariates between the treatment groups. The covariates were age group (≥65 and <65 years), BMI, duration of T2DM (≥10 years and <10 years), CVD, chronic kidney disease, hypertension, and the use of various medications and baseline biomarkers (e.g., metformin, SU_GLN, AGI, TZD, SGLT2 inhibitor insulin, angiotensin‐converting enzyme inhibitors/angiotensin receptor blockers, beta blockers, antiplatelet therapy, total cholesterol, triglycerides, high‐density lipoprotein, low‐density lipoprotein, and diastolic blood pressure). To assess the robustness of the primary endpoint evaluation—specifically, the effect between the two treatment groups—a Welch's two‐sample *t*‐test was performed to compare the mean outcomes of 3P‐MACE between the two groups. This analysis utilized the inverse probability of treatment weighting derived from the propensity score model with a caliper of 0.2. A total of 2228 patients (*N* = 1114 per group) were included in the matched analysis. The complete sensitivity analysis process is shown in Table S3.

The effect of PEG‐Loxe on the primary outcome was assessed in seven predefined subgroups (i.e., age, sex, duration of T2DM, glycated hemoglobin, BMI, history of CVD, or use of SGLT2 inhibitors)^10^ by including the subgroup and interaction term in the Cox model.

Note that *p *< 0.05 was considered statistically significant. Statistical analyses were conducted using SPSS software version 27 (IBM Corp.).

## AUTHOR CONTRIBUTIONS

K.H. and J.L. designed the trial. Y.T. and L.L. wrote the protocol. Y.Z., S.Y., W.X., J.Y., H.X., B.X., and F.D. contributed to patient recruitment and data collection. D.Z., J.C., and W.W. performed the statistical analysis. K.H. and J.L. drafted the manuscript. Y.T., Y.D., Z.H., W.L., and Z.C. revised the manuscript. All authors have read and approved the final manuscript.

## CONFLICT OF INTEREST STATEMENT

Y.D. and Z.H. are employed by Jiangsu Hansoh Pharmaceutical Group Co., Ltd. Z.C. is an editorial board member of MedComm. Z.C. was not involved in the journal's review of or decisions related to this manuscript. The other authors declare no conflicts of interest.

## ETHICS STATEMENT

The Institutional Review Board of the Second Affiliated Hospital of Shantou University Medical College approved the study protocol (Approval number: 2023–39). The study was conducted in accordance with the principles of the Declaration of Helsinki and has been registered in the China Clinical Trial Registry (ChiCTR2300073001).

## Supporting information



Supporting Information

## Data Availability

The data that support the findings of this study are available from the corresponding authors upon reasonable request.
