# Supplementary material for: Once‐weekly glucagon‐like peptide receptor agonist polyethylene glycol loxenatide protects against major adverse cardiovascular events in patients with type 2 diabetes: a multicenter ambispective cohort study (FLYING trial)
Source: MedComm (2020). 2025 Feb 13;6(2):e70094. doi: 10.1002/mco2.70094 (PMC11822460; doi:10.1002/mco2.70094)
Supplement: Supplementary file 1 — Supporting Information [file MCO2-6-e70094-s001.docx]

**Effects of once-weekly glucagon-like peptide receptor agonist polyethylene glycol loxenatide on cardiovascular outcomes in patients with type 2 diabetes: a multicenter ambispective cohort study (FLYING Trial)**

Includes:

Table S1-S6

Fig. S1

**Table S1.** The multivariate Cox regression model.

|  | Control  (n=11059) | PEG-Loxe  (n=1282) | *P* Value |
| --- | --- | --- | --- |
| 3P-MACE |  |  |  |
| No. of events | 1263 | 51 |  |
| Hazard Ratio (95% CI) |  |  |  |
| Model 1 | 1.00 (Reference) | 0.75 (0.56–0.99) | 0.048 |
| Model 2 | 1.00 (Reference) | 0.68 (0.50–0.92) | 0.013 |
| Model 3 | 1.00 (Reference) | 0.65 (0.48–0.89) | 0.006 |
| Model 4 | 1.00 (Reference) | 0.68 (0.49–0.94) | 0.019 |

HR were estimated using the following models: Model 1, adjusted for age, gender, BMI, duration; Model 2, Model 1 and further adjusted for baseline HbA1c, TC, TG, LDL-C, HDL-C, SBP, DBP, smoke, CVD history, DKD, Diabetic retinopathy ,history of hypertension; Model 3, Model 2 and further adjusted for metformin, SU/GLN, AGI, TZD, SGLT2i, Insulin, ACEI/ARB, Beta-blocker, statin, Fibrate, antiplatelet drugs; Model 4, Model 3 and further adjusted for the centers where patients were treated.

**Table S2**. Baseline Characteristics after propensity score matching.

| **Characteristic** | **PEG-Loxe**  (n=1114) | **Control**  (n=1114) |
| --- | --- | --- |
| Age (years) | 62.5 (14.3) | 60.8 (11.1) |
| Female (%) | 470 (42.2) | 487 (43.7) |
| HbA1c (%) | 8.52 (1.22) | 8.31 (3.14) |
| BMI (kg/m²) | 26.8 (1.6) | 26.9 (4.1) |
| Duration (years) | 9.7 (5.6) | 10.0 (5.9) |
| CVD history (%) | 512 (46.0) | 512 (46.0) |
| SBP, mmHg | 137.8 (16.3) | 138.3 (16.8) |
| DBP, mmHg | 80.0 (11.8) | 79.5 (11.6) |
| TC, mmol/L | 4.95 (1.04) | 4.96 (2.01) |
| TG, mmol/L | 2.17 (1.88) | 2.09 (3.57) |
| HDL-C, mmol/L | 1.20 (0.32) | 1.12 (0.48) |
| LDL-C, mmol/L | 2.92 (0.80) | 2.96 (1.19) |
| Smoke, N (%) | 123 (11.0) | 142 (12.7) |
| CKD, N (%) | 418 (37.5) | 408 (36.6) |
| Diabetic retinopathy, N (%) | 84 (7.5) | 80 (7.2) |
| History of hypertension, N (%) | 935 (83.9) | 932 (83.7) |
| Metformin, N (%) | 475 (42.6) | 450 (40.4) |
| SU/GLN, N (%) | 63 (5.7) | 49 (4.4) |
| AGI, N (%) | 411 (36.9) | 452 (40.2) |
| TZD, N (%) | 72 (6.5) | 66 (5.9) |
| SGLT2i, N (%) | 83 (7.5) | 66 (5.9) |
| Insulin, N (%) | 407 (36.5) | 407 (36.5) |
| ACEI/ARB, N (%) | 707 (63.5) | 700 (62.8) |
| Beta-blocker, N (%) | 371 (33.3) | 383 (34.4) |
| Statin, N (%) | 737 (66.2) | 753 (67.6) |
| Fibrate, N (%) | 76 (6.8) | 61 (5.5) |
| Antiplatelet, N (%) | 524 (47.0) | 534 (47.9) |

*AGI* alpha-glucosidase inhibitors, *ACEI/ARB* angiotensin-converting enzyme inhibitors/angiotensin II receptor blockers, *BMI* body mass index, *CVD* cardiovascular disease, *DBP* diastolic blood pressure, *GLN* glinides, *HbA1c* glycated hemoglobin, *HDL-C* high-density lipoprotein cholesterol, *LDL-C* low-density lipoprotein cholesterol, *PEG-Loxe* polyethylene glycol loxenatide, SBP systolic blood pressure, SGLT2i sodium–glucose cotransporter-2 inhibitors, *SU* sulphonylureas, *TC* total cholesterol, *TG* triglyceride, *TZD* thiazolidinediones.

**Table S3.** The sensitivity analysis for the primary endpoint.

| Event | PEG-Loxe  (n=1114) | Control  (n=1114) | Hazard Ratio (95% CI) | P Value |
| --- | --- | --- | --- | --- |
|  | no. of participants (%) | no. of participants (%) |  |  |
| 3P-MACE | 40 (3.6) | 166 (14.9) | 0.63 (0.43–0.91) | 0.015 |

*PEG-Loxe* polyethylene glycol loxenatide, *3P-MACE* 3-point major adverse cardiovascular events.

**Table S4.** Propensity Score Matching process

| Step 1 | **Covariates from Raw data**  1. Patient Characteristics:  - age, sex, weight (kg), BMI, smoke, HbA1c, T2DM_duration  2. Medical History:  - CKD, CVD, Hypertension, Diabetic retinopathy  3. Medication:  - T2DM: metformin, Sulfonylureas (SU)_Glinides (GLN), AGI, TZD, SGLT2i, Insulin  - CVD: ACEI/ARB, Beta-Blockers, Antiplatelet  - Lipid: Statins, Fibrates  4. Lab indicators:  - Blood Lipid: TC, TG, HDL, LDL  - Blood Pressure: SBP, DBP |
| --- | --- |
| Step 2 | **Significant Variables Selected by Stepwise**  Significant coefficients (P<0.05)  1.Patient Characteristics:  - Age group (>=65 and <65), BMI, T2DM_duration (>10 and <=10)  2.Medical History:  - CVD, CKD, hypertension  3.Medication:  - for diabetes: Metformin, SU_GLN, AGI, TZD, SGLT2i, insulin  - for CVD: ACEI/ARB, Beta blocker, Antiplatelet  4.Lab indicators:  - Blood Lipid: TC at baseline, TG at baseline, HDL at baseline, LDL at baseline  - Blood Pressure: DBP at baseline |
| Step 3 | **PS Model – 1: 1 Nearest Matching**  **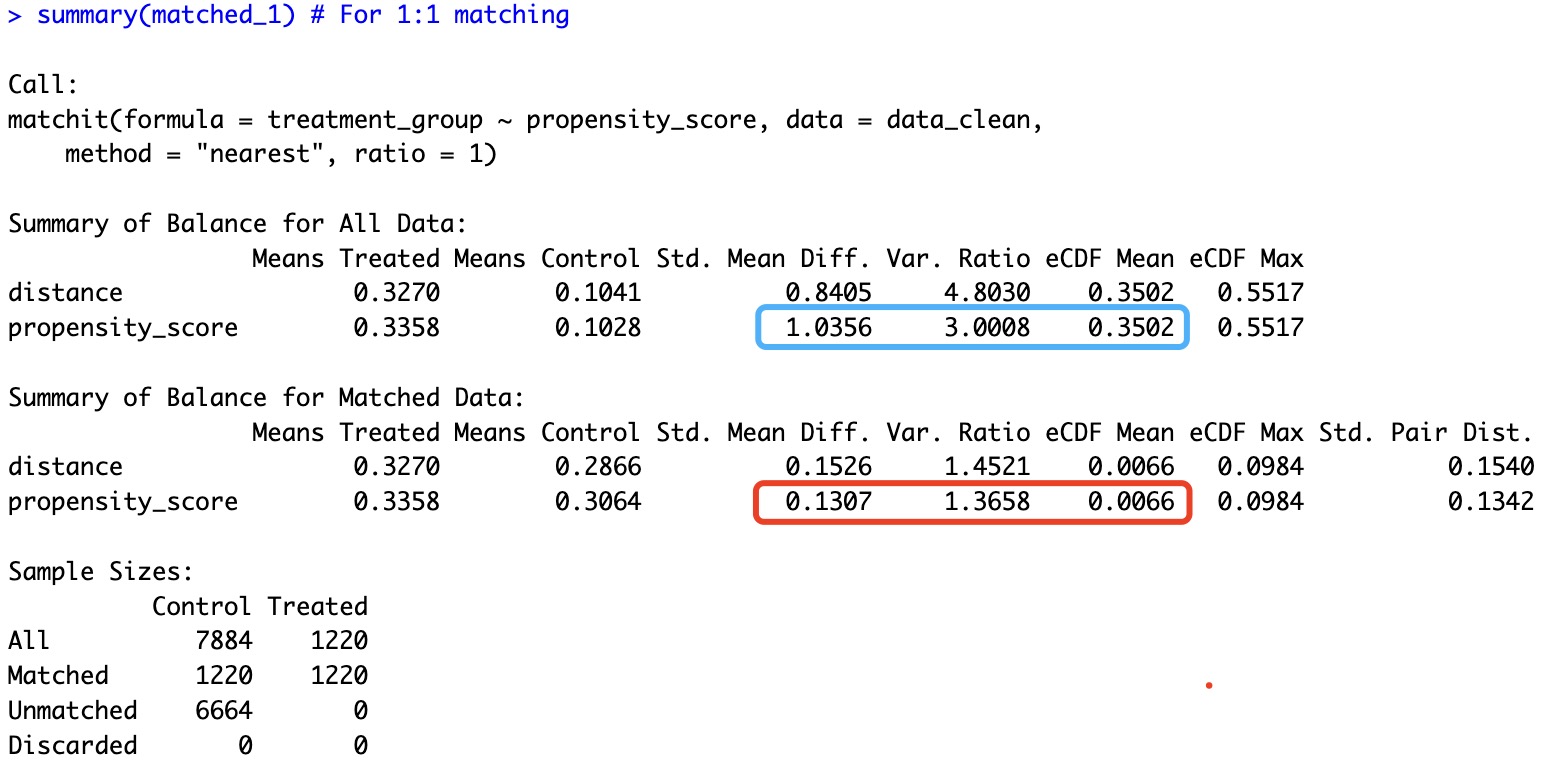** |
| Step 4 | **PS Model – 1:2 Nearest Matching**  **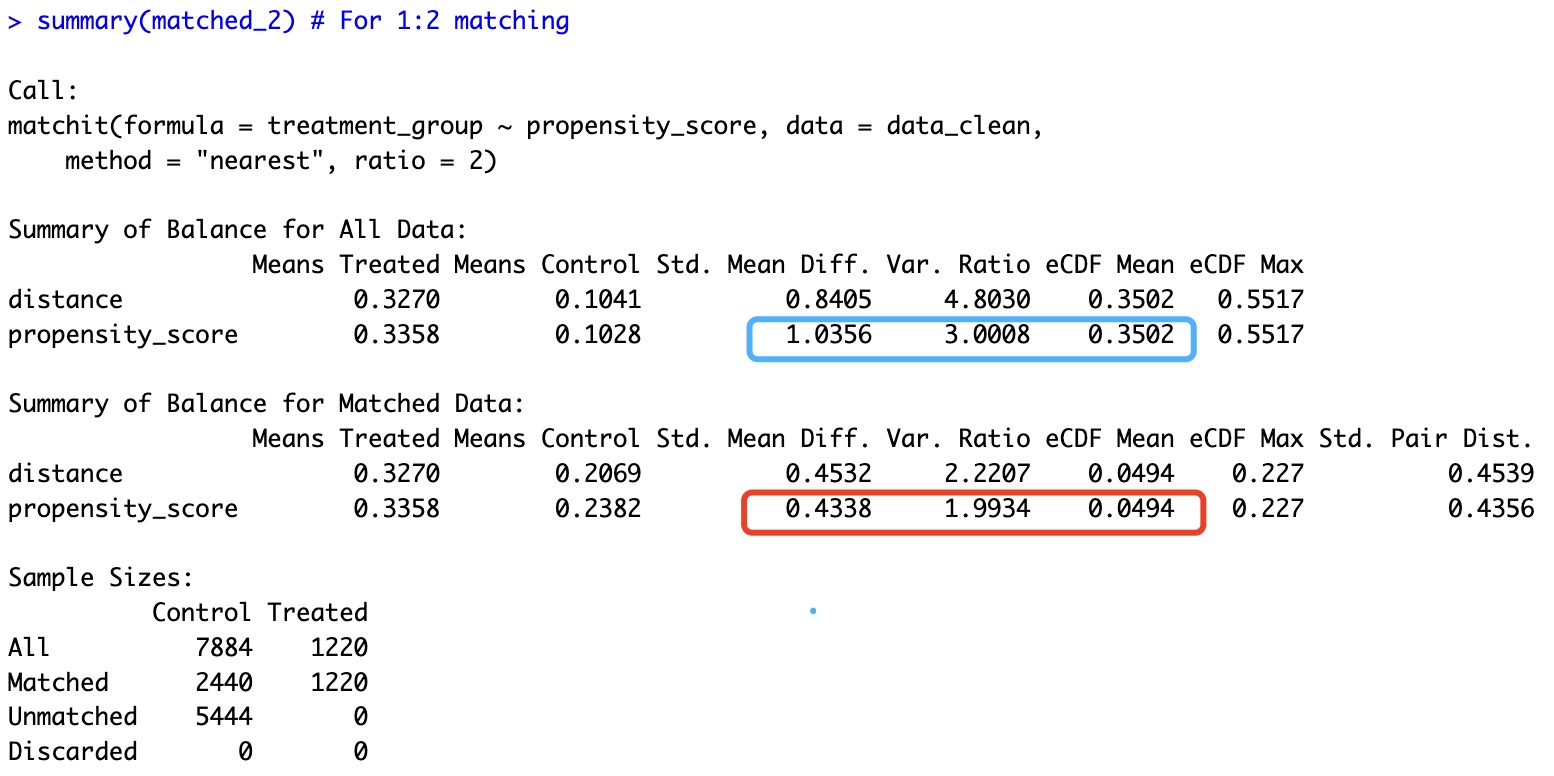** |
| Step 5 | **Love Plots for Nearest Matching**  **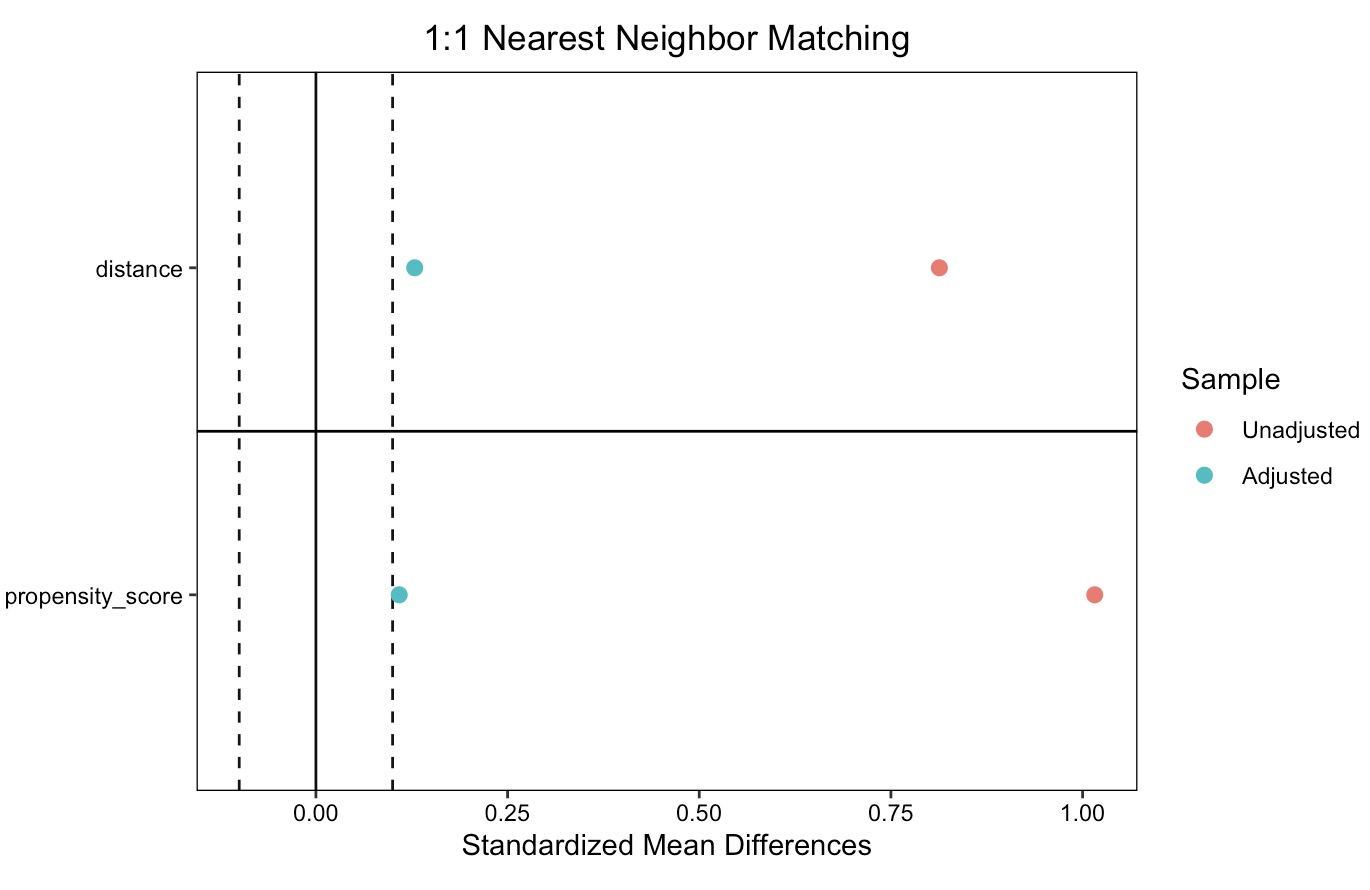**  **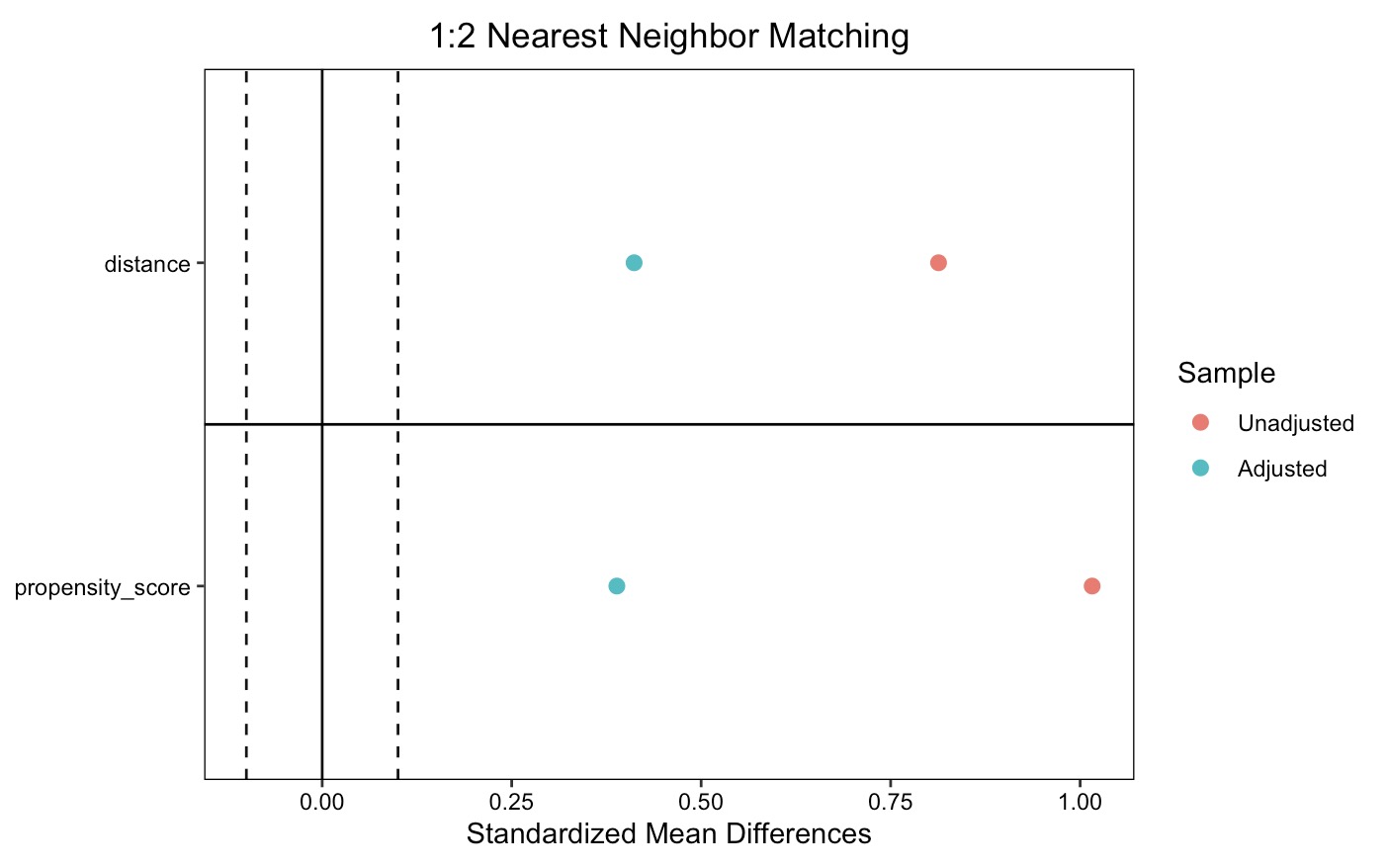** |
| Step 6 | **PS Model – Caliper=0.1 Matching**  **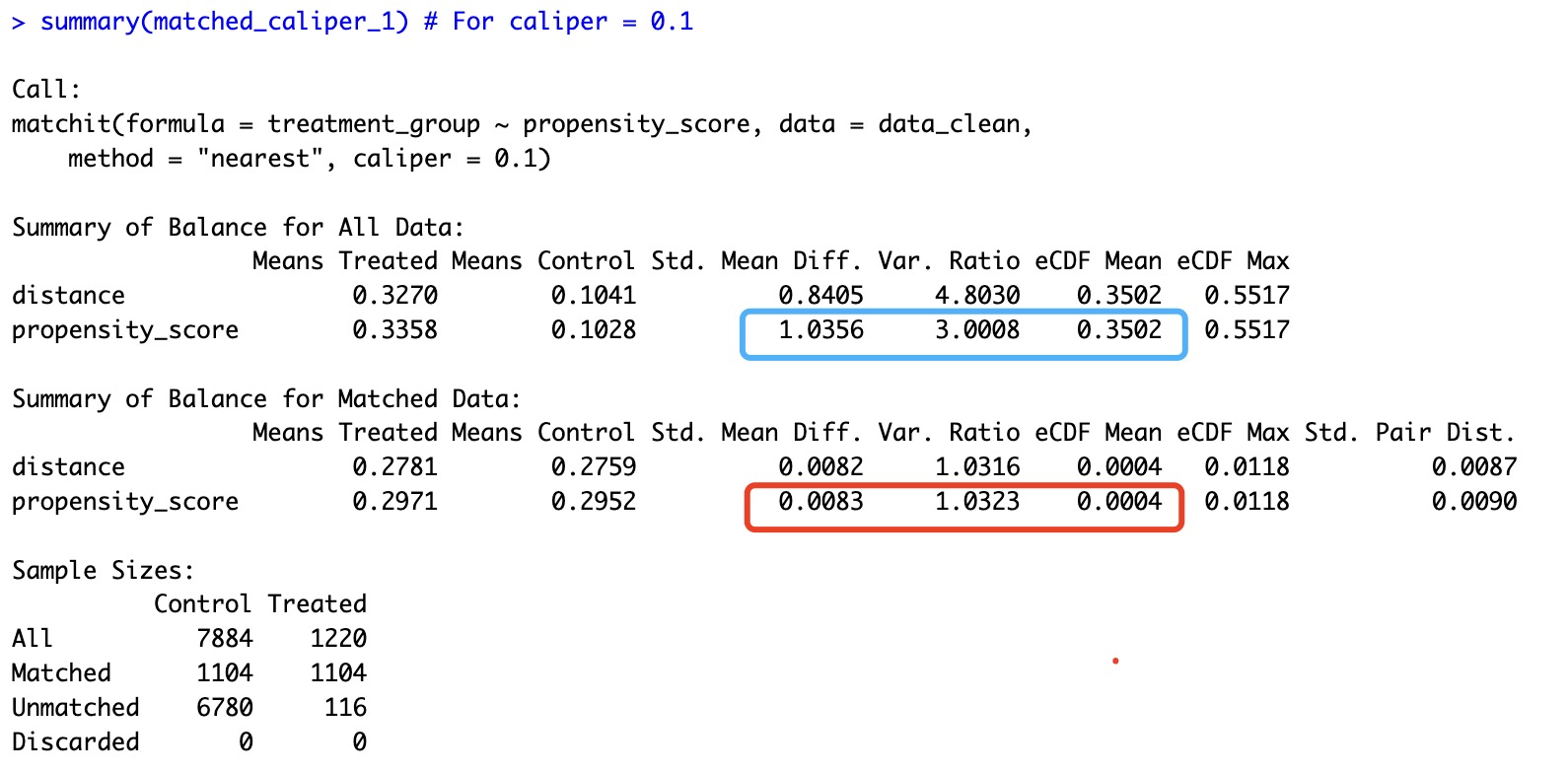** |
| Step 7 | **PS Model – Caliper=0.2 Matching**  **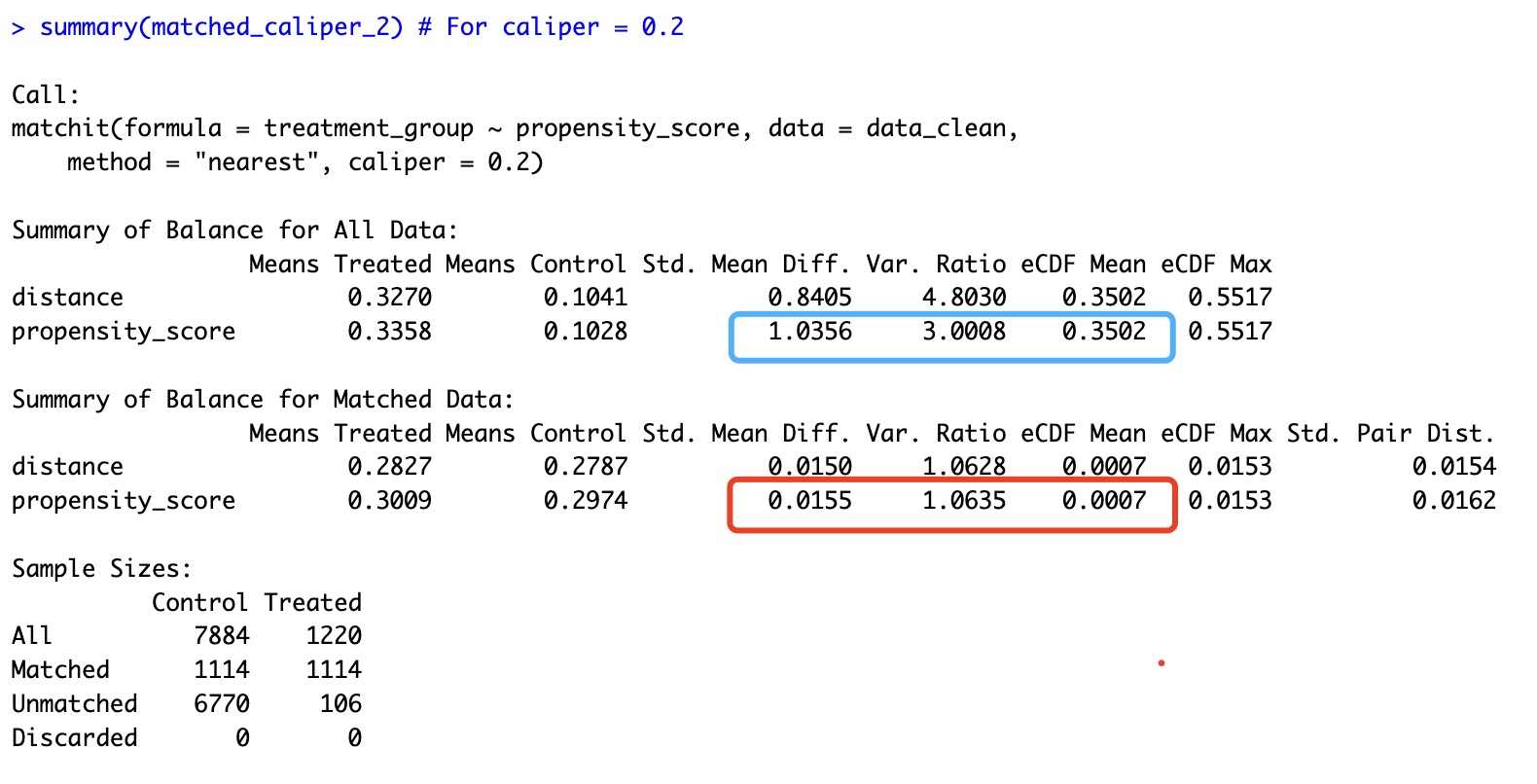** |
| Step 8 | **Love Plots for Caliper Matching**  **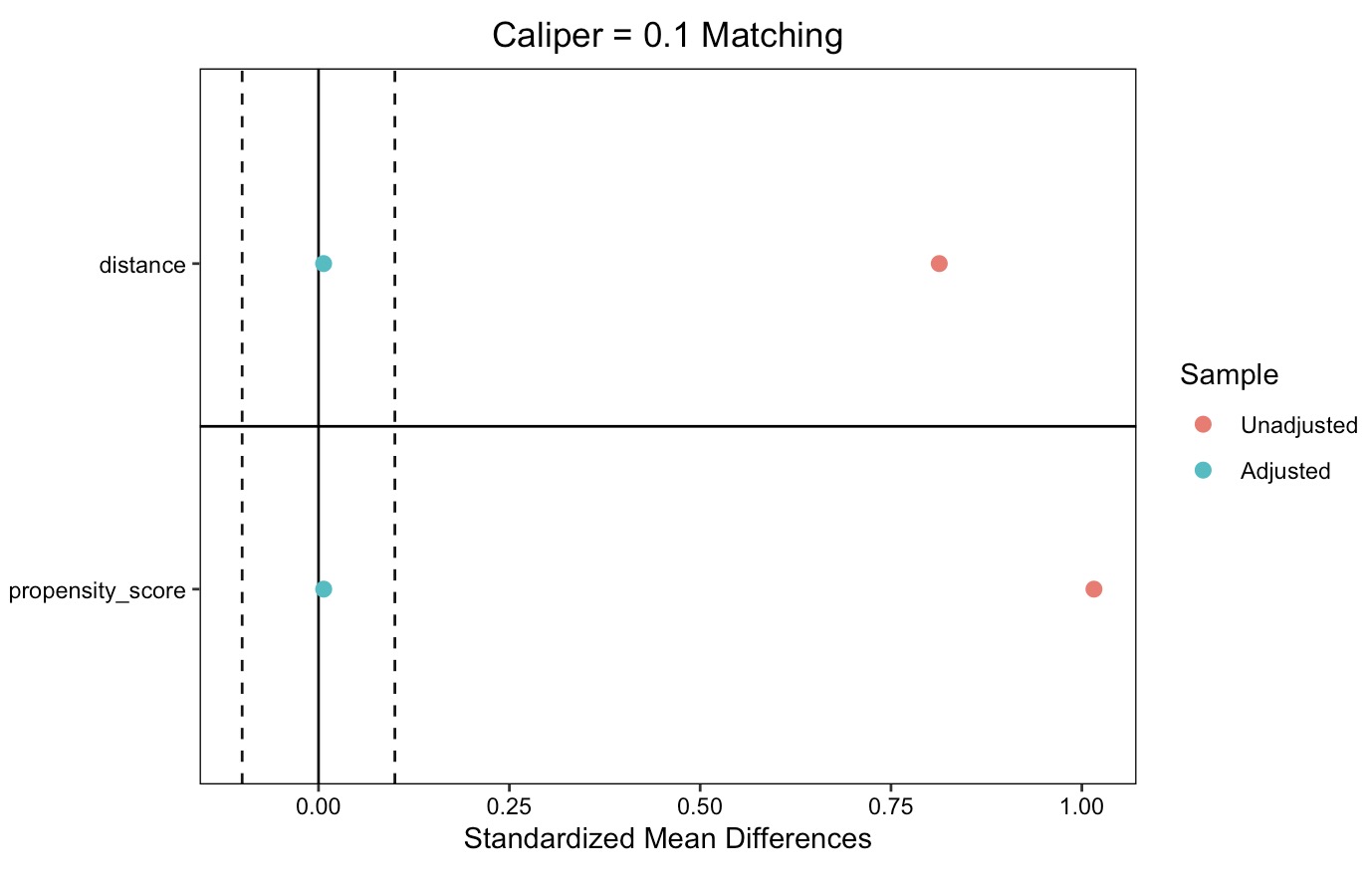**  **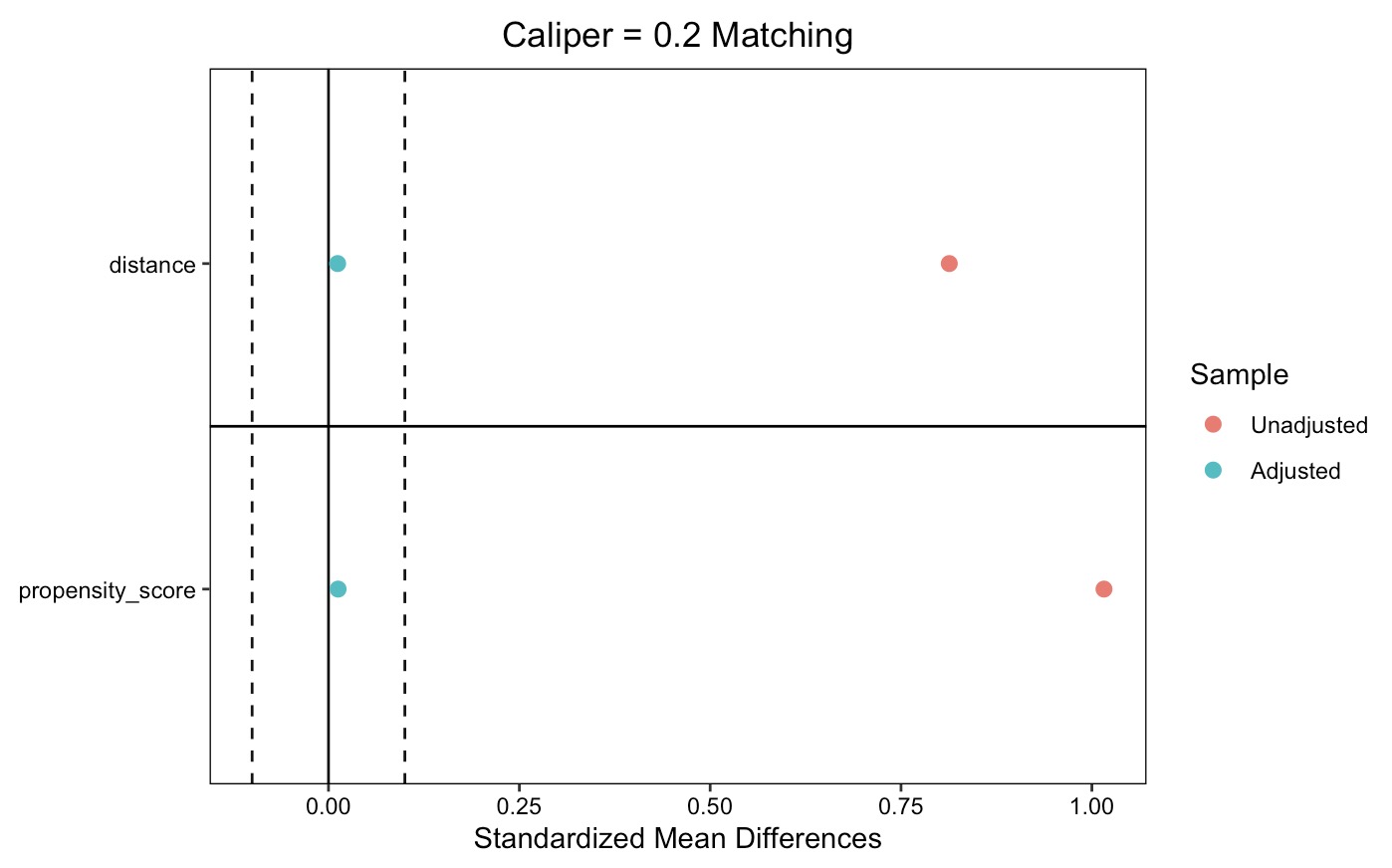** |
| Step 9 | **PS Model - Optimal Matching**  **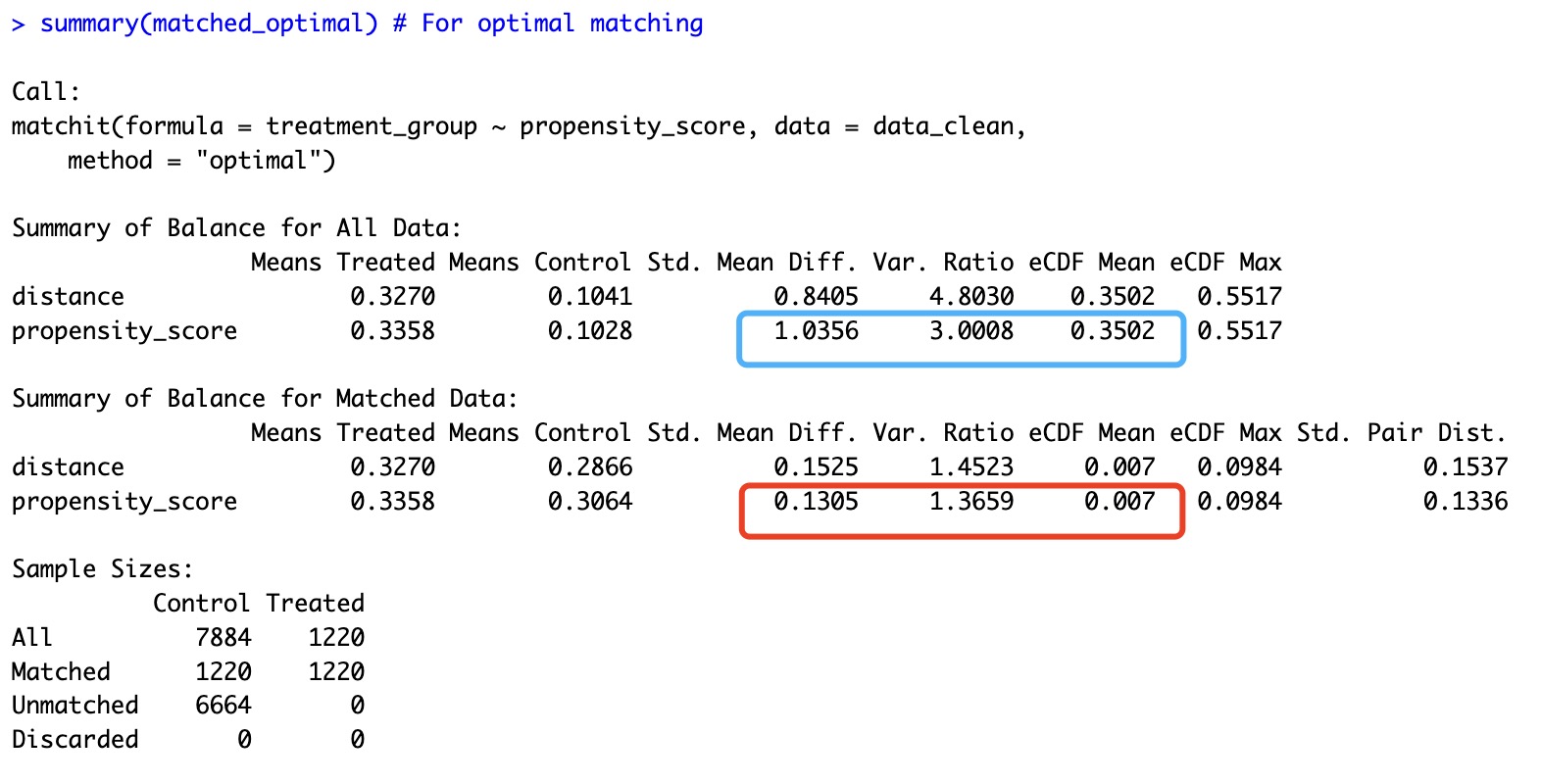** |
| Step 10 | **Love Plot Optimal Matching**  **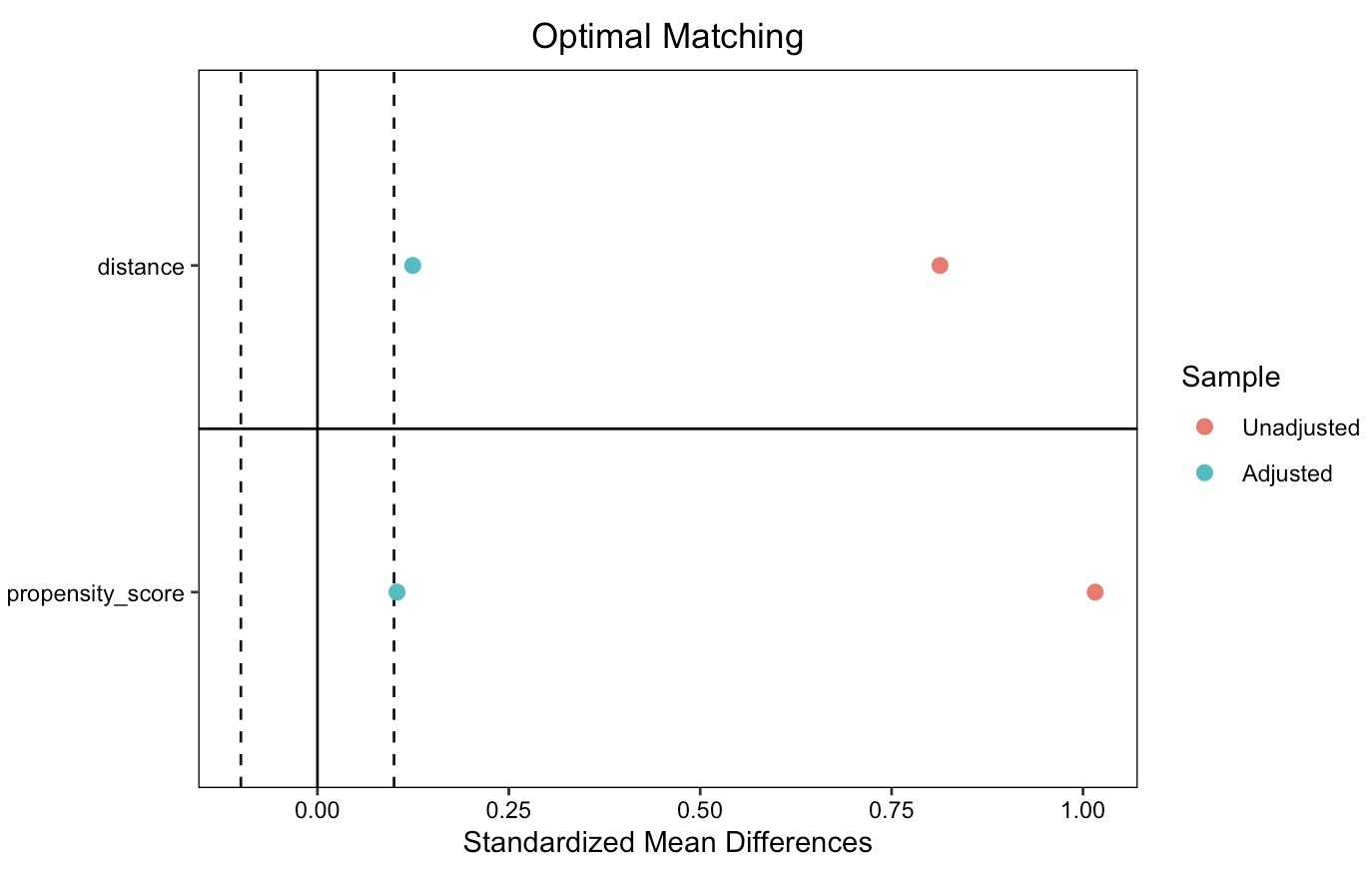**  Among the matching outcomes evaluated, the caliper = 0.2 matching approach demonstrates superior performance.  This method yields the most balanced standardized mean differences, which are closest to zero, indicating minimal bias (SDM <0.1). Additionally, it achieves the smallest variance ratios, approaching one, and exhibits the smallest empirical cumulative distribution functions (eCDFs), approaching zero. Furthermore, it maintains the maximum sample size, ensuring robustness in the analysis. |
| Step 11 | **Propensity Score Matching Comparison**  The propensity-score matching analysis was conducted to achieve balance across significant covariates between the treatment groups. The covariates included: age group (≥65 and <65), body mass index (BMI), duration of type 2 diabetes mellitus (T2DM) (≥10 years and <10 years), cardiovascular disease (CVD), chronic kidney disease (CKD), hypertension, and the use of various medications and baseline biomarkers (e.g., Metformin, SU_GLN, AGI, TZD, SGLT2i, insulin, ACEI/ARB, beta blockers, antiplatelet therapy, total cholesterol (TC), triglycerides (TG), high-density lipoprotein (HDL), low-density lipoprotein (LDL), and diastolic blood pressure (DBP)). The characteristics of the cohort matched with a caliper of 0.2 are detailed in the supplementary materials.  To assess the robustness of the primary endpoint evaluation—specifically, the effect between the two treatment groups—a Welch Two-Sample t-test was performed to compare the mean outcomes of Major Adverse Cardiovascular Events (MACE) between two groups. This analysis utilized the Inverse Probability of Treatment Weighting (IPTW) derived from the propensity score model with a caliper of 0.2. A total of 2,228 patients (N=1114 per group) were included in the matched analysis. |
| Step 12 | **Welch Two Sample t-test**  **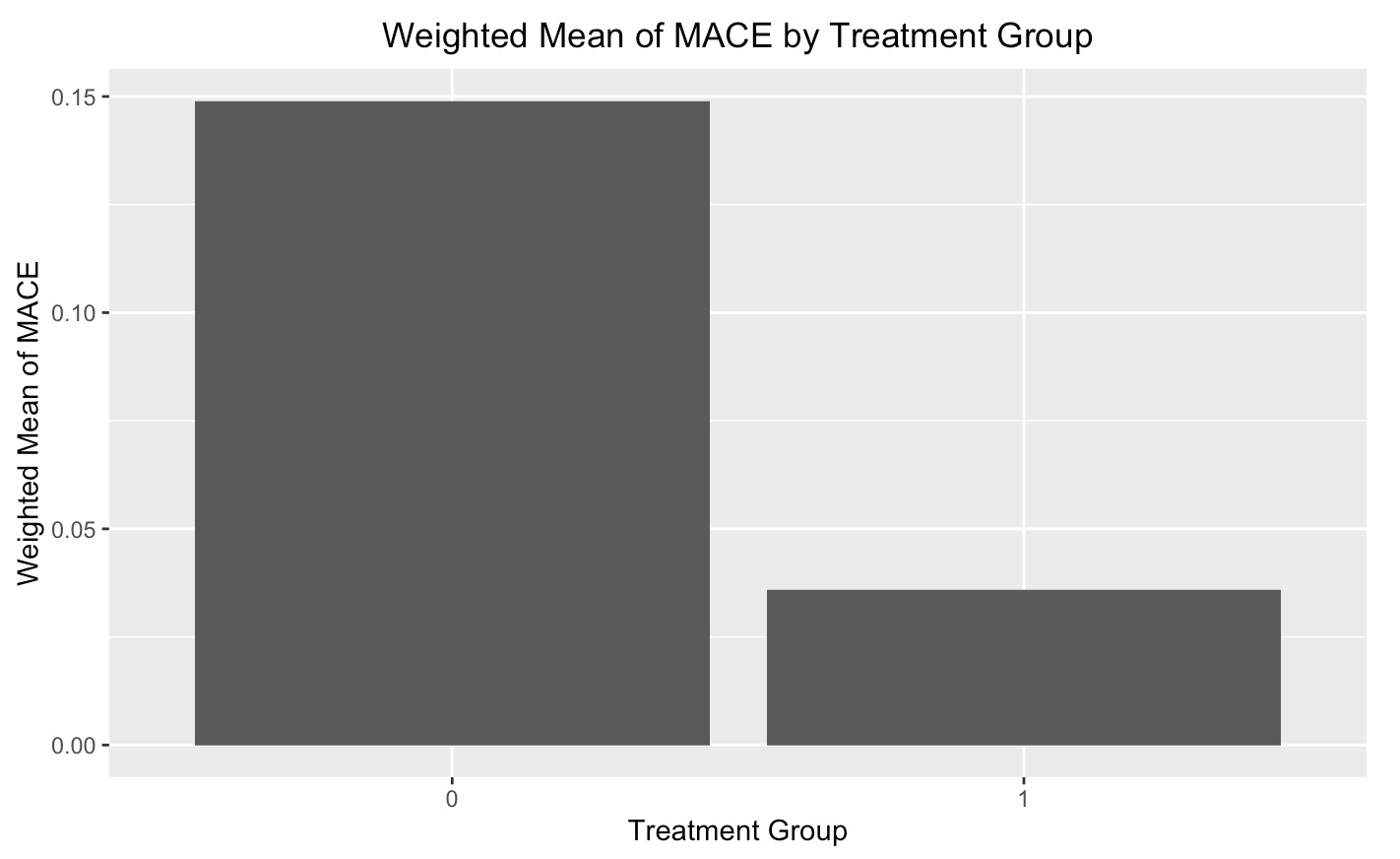**  **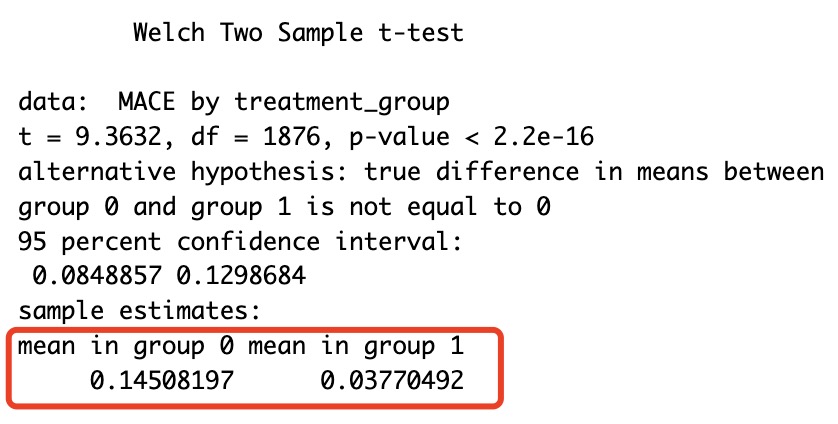**  The Welch Two Sample t-test indicates a statistically significant difference in the mean MACE between two treatment groups (p < 2.2e-16). These results support the conclusion that there is a significant difference in the occurrence of MACE between the two treatment groups. |
| Step 13 | **Adjusted Multivariate Cox Model**  Treatment Group:  Coefficient: -0.4642, Hazard Ratio (HR): 0.6287, p-value: 0.0147  The PEG-Loxe is associated with a 37.1% reduction in the hazard of experiencing Major Adverse Cardiovascular Events (MACE) compared to the control group. |

**Table S5**. Cardiovascular disease or cardiovascular risk factor.

| Cardiovascular disease^1^ | MI history  History of coronary, carotid or peripheral artery revascularization  >50% vascular stenosis with imaging of the coronary, carotid or lower extremity arteries  ECG or cardiac imaging demonstrating a history of stable coronary artery disease  History of stroke or transient ischemic attack  Ankle/brachial index <0.9 |
| --- | --- |
| Cardiovascular risk factor^1^ | BMI ≥32.5 kg/m2  Dyslipidemia despite lipid-lowering treatment  Tobacco use  UACR ≥30 mg/g  SBP >140 mm Hg or DBP >90 mm Hg despite antihypertensive medication  A family history of early coronary heart disease in a 1st-degree relation (male relatives aged <55 years old or female relatives aged <65 years old) |

Reference ^1^: Xie, Y. et al. Impact of polyethylene glycol loxenatide on cardiovascular outcomes in patients with type 2 diabetes: study protocol for a multicentre, randomised, double-blind, placebo-controlled trial (BALANCE-3). BMJ Open 13, e069080 (2023).

**Table S6**. Definition of study endpoints.

| Myocardial infarction | ICD-10, I21 |
| --- | --- |
| Stroke | ICD-10, I60–I64 |
| Cardiovascular death | ICD-10, I00–I99 |
| Hospitalization for unstable angina | ICD-10, I20 |
| Hospitalization for heart failure | ICD-10, I50 |
| Serious gastrointestinal events^1^ | Gastrointestinal events resulting in death, initial or prolonged in-patient hospitalization, persistent or significant disability or incapacitation, or congenital abnormality or birth defect, life-threatening events, and any other event considered serious by the investigator. |
| Diabetes-related blindness^2^ | blindness due to diabetic retinopathy |
| Acute pancreatitis | ICD-10, K85 |
| Cancer | ICD-10, C00-C97 |
| Medullary thyroid carcinoma^3^ | Medullary thyroid carcinoma is an uncommon malignancy of neuroendocrine origin derived from the parafollicular C cells. |
| Acute kidney failure | ICD-10, N17 |
| Severe hypoglycemia^4^ | Severe hypoglycemia is defined as a condition with serious cognitive dysfunction requiring external help from other persons. |


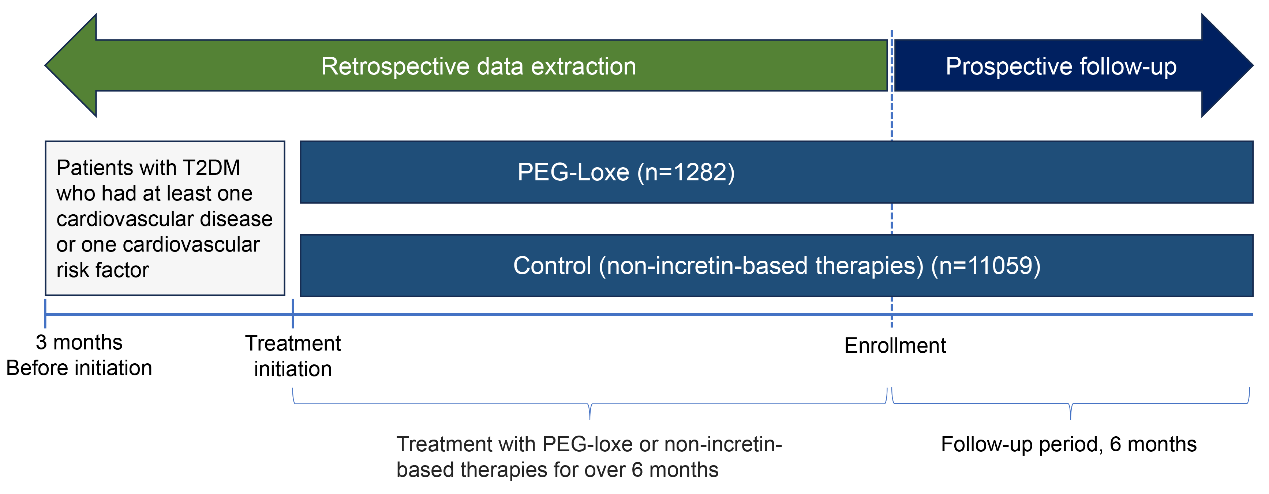


**Fig.S1** Study design.

**REFERENCE**

1. Horowitz M, et al. Upper and/or lower gastrointestinal adverse events with glucagon‐like peptide‐1 receptor agonists: Incidence and consequences. Diabetes Obes Metab. 2017;19(5):672-681.

2. Scanlon PH. The contribution of the English NHS Diabetic Eye Screening Programme to reductions in diabetes-related blindness, comparisons within Europe, and future challenges. Acta Diabetol. 2021;58(4):521-530.

3. Ravindri Jayasinghe, et al.Management of medullary carcinoma of the thyroid: a review. J Int Med Res. 2022;50(7):3000605221110698.

4. Urakami T. The Advanced Diabetes Technologies for Reduction of the Frequency of Hypoglycemia and Minimizing the Occurrence of Severe Hypoglycemia in Children and Adolescents with Type 1 Diabetes. J Clin Med. 2023;12(3):781.
